# Fibrosing Mediastinitis Presenting With Superior Vena Cava Syndrome

**DOI:** 10.7759/cureus.23700

**Published:** 2022-03-31

**Authors:** Jee Ha Park, Jon Lucaj, Krassimir L Denchev

**Affiliations:** 1 Anesthesiology, Wayne State University School of Medicine, Detroit, USA

**Keywords:** mediastinitis management, re-vascularization, critical care, clinical case report, superior vena cava (svc) syndrome, fibrosing mediastinitis

## Abstract

Fibrosing mediastinitis (FM) is an uncommon diagnosis that involves the proliferation of extensive, dense fibrous tissue in the mediastinum. FM accounts for less than 1% of people with prior infection by *Histoplasma capsulatum *that develop hypersensitivity immune responses to antigens released during the initial exposure. Other causes include tuberculosis, blastomycosis, sarcoidosis, radiation, and idiopathic.

We describe FM presenting with superior vena cava (SVC) syndrome. A 66-year-old Caucasian male presented with a one-week history of progressively worsening facial swelling associated with dysphonia, bilateral ptosis, dyspnea on exertion, and unintentional weight loss of 30 pounds within the past three months. He had a 40-pack-year smoking history and a past medical history of essential hypertension, peripheral vascular disease, and bilateral internal carotid artery stenosis. The CT chest demonstrated non-specific soft tissue extending throughout the mediastinum and towards the right hilar region, complicated by severe attenuation of the superior vena cava and a 2.4 cm × 1.6 cm necrotic lymph node. The mediastinum had hyperemic and desmoplastic changes heavily encased in venous collaterals. L4 lymph node pathological evaluation demonstrated sinus histiocytosis and reactive lymphoid hyperplasia without signs of malignancy or atypia. The patient was treated with corticosteroid and diuretic therapy to achieve intermittent symptomatic relief, but continued to decline clinically, ultimately leading to his demise.

The diagnosis of FM is best obtained through CT chest with intravenous contrast to demonstrate abnormal mediastinal tissue and possible structural compromise. A biopsy of the mediastinal tissue may also help rule out malignancy. Only a few case reports have demonstrated mixed symptomatic and radiologic responses to anti-inflammatory and/or antifungal treatment. Even non-surgical and surgical interventions have shown inconsistent efficacy, with frequent restenosis warranting re-exploration.

## Introduction

Fibrosing mediastinitis (FM) is a rare disease and has been a part of the National Organization of Rare Disorders since 2007. It is characterized by a local expansion of fibroinflammatory tissue within the mediastinum. Patients present with non-specific signs such as fever, fatigue, dyspnea, cough with or without hemoptysis, chest pain, and superior vena cava (SVC) syndrome. Much of the symptoms are a result of external obstruction of mediastinal structures (SVC, pulmonary arteries/veins, central airways, etc.) by the fibrous tissues; therefore, patients do not develop symptoms until a degree of degeneration has occurred in the normal anatomy. No precise pathophysiology of FM has been described; however, it has been thought to be caused by Histoplasma capsulatum or idiopathic etiologies.

In North America, FM is often associated with H. capsulatum as a rare hyperactive immunologic complication affecting only 1% of those with a history of histoplasmosis. For example, only three out of 100,000 people with acute histoplasmosis demonstrated FM in an outbreak in Indianapolis [1]. 80% of FM has been postulated to be related to histoplasmosis [2]. Acute histoplasmosis can result in granulomatous mediastinitis, characterized by granulomas on tissue biopsy; however, there are no data that granulomatous mediastinitis develops into FM [3].

FM has also been associated with a more systemic inflammatory process such as immunoglobulin G4-related disease (IgG4RD) [4,5]. One-third of patients demonstrated FM as a part of systemic multiorgan fibrosis in IgG4RD [5]. This association emphasized the likely role immunologic responses play in the development of FM. Other etiologies include tuberculosis, blastomycosis, sarcoidosis, radiation, and idiopathic diseases.

SVC syndrome refers to clinical signs and symptoms that occur secondary to obstruction of SVC venous outflow. The obstruction can occur intra or extra-vascular. The etiologies can largely be divided into malignant and benign causes. Malignant causes account for 60% [6] of SVC syndrome cases and include small cell lung carcinoma, non-small cell lung carcinoma, non-Hodgkin’s lymphoma, and metastatic carcinoma. The most common benign cause is iatrogenic thrombus or stenosis secondary to prolonged intravascular catheter placement. Other benign etiologies include radiation, autoimmune, and vascular pathologies. 

This case report is of fibrosing mediastinitis presenting with SVC syndrome complemented with a review of literature on its diagnostic characteristics and treatment modalities.

## Case presentation

A 66-year-old Caucasian male from an area with a large chicken farming industry presented to the hospital with a one-week history of progressively worsening facial swelling. The patient’s chief complaint was associated with dysphonia, bilateral ptosis, and dyspnea on exertion; however, he did not demonstrate any current or future airway compromise or signs of elevated intracranial pressure. The patient also reported an unintentional weight loss of 30 pounds within the last three months. The patient had a past medical history of essential hypertension, hyperlipidemia, bilateral carotid artery stenosis, peripheral vascular disease, and a remote history of transient ischemic attack. He was worked up extensively at a different hospital system for his peripheral vascular disease but was never followed up. He was a self-proclaimed herbal enthusiast and did not take any medications. Other relevant history included a 40-pack-year smoking history, which he stopped a few years back.

**Figure 1:**
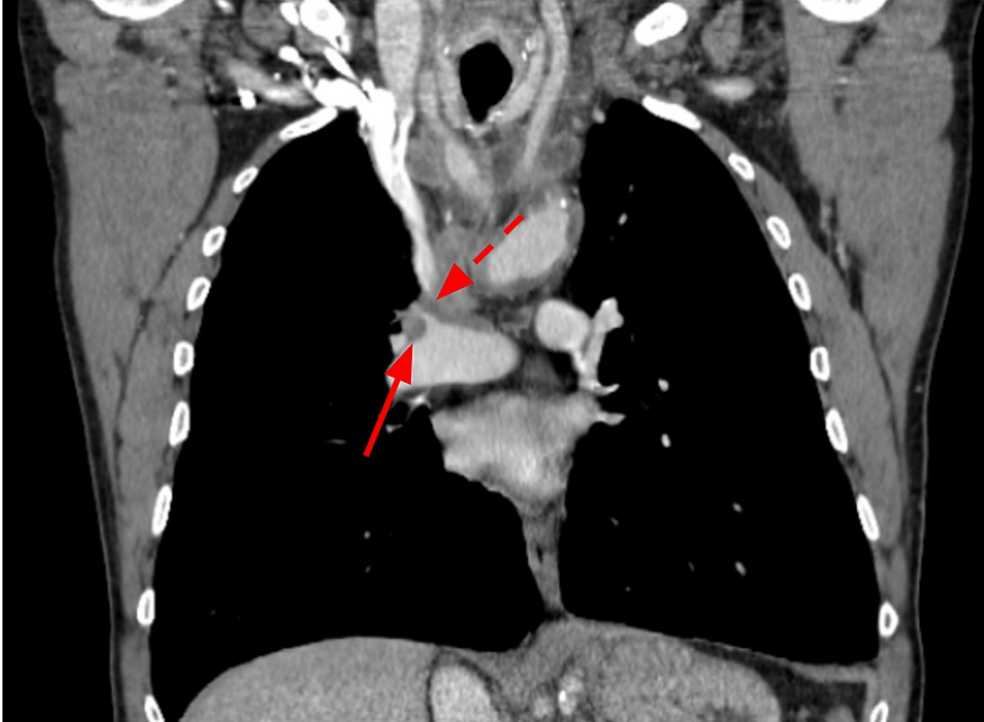
Computed tomography chest showing attenuation of the right pulmonary artery (arrow) and superior vena cava (dotted arrow).

**Figure 2:**
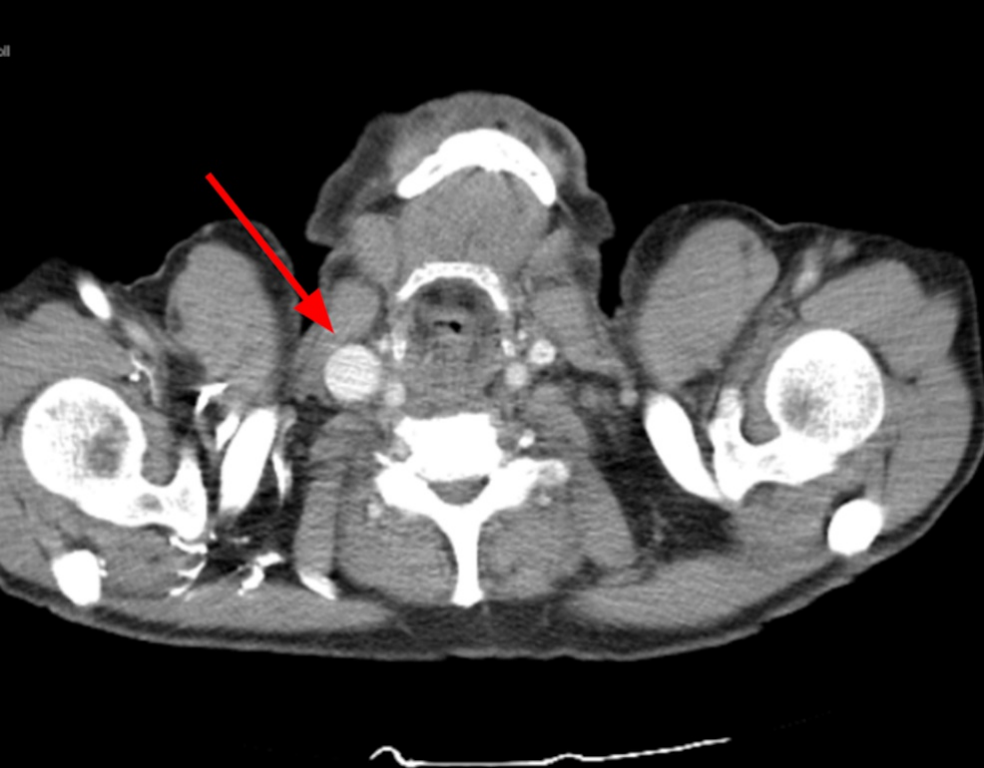
Computed tomography chest showing dilation of the right internal jugular vein (arrow) in comparison to the left internal jugular vein.

The laboratory workup was unremarkable. Initial computed tomography (CT) chest showed non-specific soft tissue extending throughout the mediastinum and towards the right hilar region in the prevascular and paratracheal space that severely attenuated the superior vena cava as it entered the right atrium and some of the right upper lobe pulmonary artery (Figure 1). There was also a 2.4 cm × 1.6 cm necrotic lymph node in the prevascular space in the upper trachea, concerning lymphoma. The right internal jugular vein was distended in comparison to the left internal jugular vein (Figure 2). The CT chest also showed suggestions of non-occlusive thrombus within the left brachiocephalic vein. The CT abdomen demonstrated complete occlusion of infrarenal aortic stenosis with significant arterial collaterals as well as moderate focal stenosis in the superior mesenteric and celiac artery; however, the patient did not show signs of abdominal symptoms or arterial insufficiency on presentation. Magnetic resonance imaging of the brain ruled out brain metastasis. Imaging findings confirmed the diagnosis of SVC syndrome.

**Figure 3:**
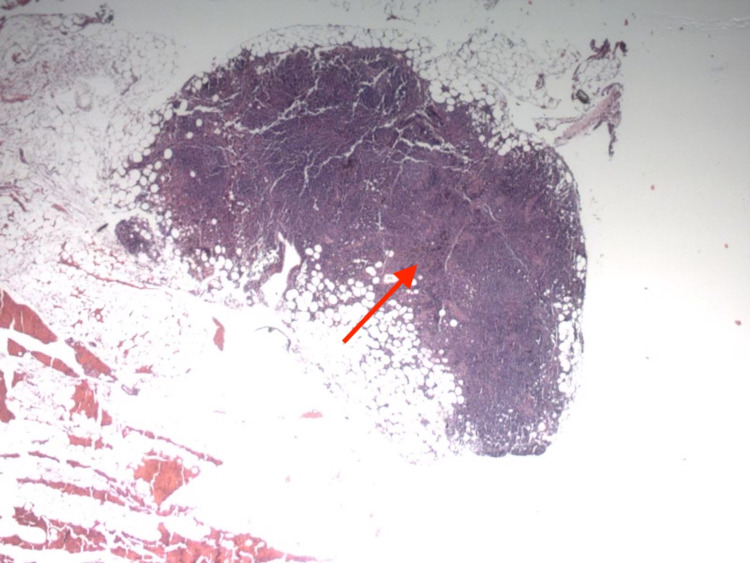
Hematoxylin and eosin staining of the right L4 lymph node. Sinus histiocytosis and reactive lymphoid hyperplasia without signs of malignancy or atypia (arrow).

The patient’s SVC syndrome was symptomatically treated with furosemide and intravenous dexamethasone. The patient demonstrated symptomatic relief from facial swelling with initial treatment. The patient was hemodynamically stable on presentation, and our initial goal in management was to rule out malignancy. The patient was taken to the operating room for diagnostic mediastinoscopy with lymph node biopsy, which was converted to video-assisted thoracoscopy. Upon visualization, the mediastinum was heavily encased in venous collaterals with desmoplastic changes and mediastinal pleura with significant hyperemia. There was SVC induration without an overt mass, suggesting the diagnosis of FM. An R4 lymph node was sampled for pathology evaluation. Pathology evaluation reported sinus histiocytosis and reactive lymphoid hyperplasia without signs of malignancy or atypia (Figure 3). With our findings from the patient's environmental exposure, CT chest imaging, surgical visualization, and pathology evaluation, the diagnosis of FM was confirmed to be the etiology of the patient's SVC syndrome.

After lymphoma had been ruled out with surgical biopsy, the patient’s SVC syndrome was managed symptomatically with continued dexamethasone and furosemide. Facial swelling persisted with intermittent improvements only when the diuretic dose was increased, with overall worsening. The patient was treated symptomatically; however, the disease itself was unable to be controlled.

The patient developed airway edema as a consequence of the SVC syndrome, leading to hypercapnic hypoxic respiratory failure requiring intubation and ventilatory support. The hospital course was further complicated by underlying arterial disease. He developed severe lactic acidosis due to occlusive peripheral arterial disease and bowel ischemia, requiring multiple vasopressors and inotropic support. Unfortunately, complications from his arterial and venous disease resulted in his demise.

## Discussion

Diagnosis 

There are no exact criteria for diagnosing FM; clinically, patients present with non-specific symptoms. The initial step to diagnosis is to rule out malignancy. Imaging is crucial for the exclusion of underlying malignancy, assessment of disease progression, identification of structural compromise, and evaluation of treatment response; however, a surgical biopsy may be required to completely rule out malignancy. Laboratory tests can further differentiate FM from other mediastinal pathologies [7].

Two distinct radiologic patterns of FM have been described in a retrospective study of 33 patients with FM. 82% of patients demonstrated localized patterns, often with calcification (63%), and 18% of patients demonstrated diffuse patterns [8]. It has been proposed that the localized and diffuse patterns are associated with histoplasmosis and idiopathic causes of FM, respectively.

Histological evaluation of FM, in general, demonstrates inflammatory fibrous tissue with infiltration of mononuclear cells. A three-stage staging system has been proposed based on histopathologic evaluation: stage I demonstrates fibromyxoid tissue with numerous immune cells; stage II demonstrates thick eosinophilic collagen with spindle cells and lymphocytes; and stage III, at its final stage, demonstrates acellular collagen with lymphoid follicles and dystrophic calcification [9]. The staging system gives further insight into the fibroinflammatory pathogenesis involved in FM.

Treatment modalities

Currently, no clear treatment guidelines are in place for FM. Much of the literature on the treatment of FM is confined to case reports and case series. Individual case reports have demonstrated mixed symptomatic and radiologic responses to anti-inflammatory and/or antifungal treatment. However, larger case series have failed to demonstrate similar efficacy. Non-surgical and surgical interventions to relieve mediastinal compression have also demonstrated highly variable results (Table 1).

**Table 1:** Summary of treatment modalities.

Medical	
Anti-inflammatory (corticosteroid)	May control the inflammatory process. Mixed success with unclear efficacy.
Anti-fungal	FM is not an acute fungal infection. Mixed success with unclear efficacy.
Interventions	
Non-surgical	Balloon angioplasty with or without stenting. Good initial symptom relief but a high rate of reintervention.
Surgical	Perioperative risk Resection vs. vascular reconstruction. Good long-term symptomatic relief.

Medical therapies

Anti-Inflammatory Treatment

One potential method of treatment is to control the underlying inflammatory process of FM. Isolated case reports have demonstrated symptomatic improvement with anti-inflammatory treatment for patients demonstrating compressive symptoms [10-12]. Symptomatic relief with corticosteroid therapy was consistent with radiologic improvements as well [7]. However, in larger case series, corticosteroid therapy did not demonstrate consistent results. Only one out of six patients demonstrated benefit from corticosteroid therapy, who died as a consequence of worsening pulmonary artery compression and pulmonary hypertension [5].

Given the current understanding of the pathological findings of FM, corticosteroid therapy may be a reasonable initial treatment to control the underlying fibroinflammatory process of FM. Anti-inflammatory therapy is a relatively safe treatment modality. In fact, it is the mainstay of therapy for malignant causes of SVC syndrome. However, in the case of FM, there are mixed results on the efficacy of corticosteroid therapy on the compressive symptomology of FM. With the current literature, no conclusion can be made on the role of anti-inflammatory therapy; a larger study is needed to make a more definitive conclusion.

Antifungal

It was once theorized that rupture of a mediastinal granuloma from acute histoplasmosis caused an antigenic response, causing fibrosis and FM [13,14]. This theory has largely been refuted by Dunn et al. after reviewing the literature at that time. Stemming from this idea, systemic antifungals have been proposed to stabilize FM [14]. 

Itraconazole has demonstrated success in treating FM in isolated case reports [15,16]. However, in a larger case series, only three of 34 patients demonstrated radiographic improvement, with only one of the three reporting improvement in symptoms [5]. The most widely accepted theory of FM is unrelated to an acute histoplasmosis infection and more related to a chronic antigenic reaction. Therefore, it is difficult to see the role of antifungal therapy in treating FM without a larger prospective and randomized trial.

Interventions

Much of the symptomology of FM is due to the mass effect on nearby vasculature and airways; therefore, more invasive management may be warranted for the disease. A non-surgical option is endovascular balloon angioplasty with or without stenting, whereas surgical resection of the mass and/or vascular reconstruction may be an option for better surgical candidates. The therapeutic effect of non-surgical or surgical interventions varies widely, ranging from complete resolution to failure. Even in successful cases, restenosis and re-exploration are often warranted. Another factor to consider in patients who are candidates for an intervention is the innate risk of invasive treatments and the added risk of perioperative mortality.

Non-surgical Interventions

The efficacy and the rate of recurrence of endovascular interventions are not well documented and vary amongst different reports. In a larger case series, 87%, equating to 77 patients, receiving endovascular interventions, obtained symptomatic relief; however, 28% required restenting, whether it was due to rapidly growing fibrous tissue, stent thrombosis, or tissue collapse, with a median recurrence of 115 months [17]. In another smaller case series, four patients received percutaneous balloon angioplasty with stenting and achieved hemodynamic stability. However, two out of four patients required reintervention in the first year due to in-stent thrombosis [18]. Peikert et al. studied 13 patients receiving endovascular interventions. Eleven achieved symptom relief, but eight patients developed stent restenosis with symptoms, requiring reintervention within the first year [5]. 

Non-surgical interventions allow for safer management of obstructive symptoms of FM compared to surgical options, especially in the short term. However, recurrence of symptoms and failure of the stent to remain patent pose a clinical problem. This can be attributed to the continued fibrous tissue growth of FM. Patients may require adjuvant medical therapy to slow down or eliminate continued growth. Non-surgical interventions also pose a technical challenge to the operator; higher pressure may be required to deploy the stent, and as a result, the stent can move to an area without compromise.

Surgical Interventions

Surgical options can largely be divided into excision of tissue via pneumonectomy, lobectomy, and surgical decompression and vascular reconstruction using saphenous spiral vein graft, polytetrafluoroethylene (PTFE), or Dacron graft. Invasive surgical options inherently come with a higher risk of perioperative complications and mortality. Given the underlying risk of surgery, a careful assessment of pathological anatomy must be done prior to surgery. The decision to proceed with surgery should be assessed individually.

Exploratory mediastinotomy can help assess the extent of the disease, as in this case. However, depending on how extensive of an invasion has occurred, excision of fibrous tissue carries a high risk and complexity. Pneumonectomy and lobectomy can be done on patients with non-functional lung parenchyma for good symptomatic relief, but carry high surgical complications. Three FM cases underwent pneumonectomy; two out of three patients developed bronchopleural fistula [14]. Mathisen and Grillo reported 20 cases of FM, of which 18 received surgical interventions; however, 75% of patients ended up dying due to complications of the procedure [19].

Surgical vascular reconstruction techniques are an alternative to the endovascular procedure, especially in younger patients who are safer surgical candidates. Similar to endovascular stent placement, restenosis of the graft is of concern. Peikart et al. performed 17 surgical interventions on patients with FM, with 94% of patients achieving symptomatic relief. However, four patients had surgical complications, and five patients required reinterventions, mostly due to graft stenosis [5]. In a separate case series, it reported 16 patients who received spiral saphenous vein grafts to relieve SVC syndrome caused by fibrosing mediastinitis. They reported patency in 87.5% of patients, with a mean clinical follow-up of 10.9 years after spiral graft placement [20].

Surgical interventions offer the benefit of long-term symptomatic relief with a reduced rate of reintervention. However, there is a perioperative risk that needs to be considered. Each patient needs to be assessed individually, and the risk versus benefit of surgery needs to be carefully thought out before proceeding.

## Conclusions

There is no clear guidance on the management of FM. Current data on various treatment modalities are largely based on isolated case reports and case series without a decisively superior treatment method. A large part of the uncertainty is rooted in the lack of true understanding of the pathophysiology of the disease, especially when predicting the progression and prognosis of the disease. Obtaining a fuller understanding of the different etiologies of FM may assist in alleviating the inconsistency in the results. Unfortunately, given the rarity of the disease, it may be initially difficult to obtain enough information. Further studies need to be conducted to create more robust guidance on treatment strategies for patients with FM.
